# Correction

**DOI:** 10.1111/cas.15614

**Published:** 2022-12-15

In an article^1^ titled ‘Chromobox homolog 8 is a predictor of muscle invasive bladder cancer and promotes cell proliferation by repressing the p53 pathway’ by Gang‐jun Yuan, Xin Chen, Jun Lu, Zi‐hao Feng, Si‐liang Chen, Ri‐xin Chen, Wen‐su Wei, Fang‐jian Zhou and Dan Xie, there were errors in Figures 2 and 5.

In Figure 2, the IHC image of 4 N in 2c, and in Figure 5, the Western blotting images of CBX8, GAPDH in BIU cells in 5b right and CDK4, cdc25A in 5e were inadvertently misused in the original published manuscript. The correct Figure 2c and Figures 5b and 5e are displayed below.

**Figure:**
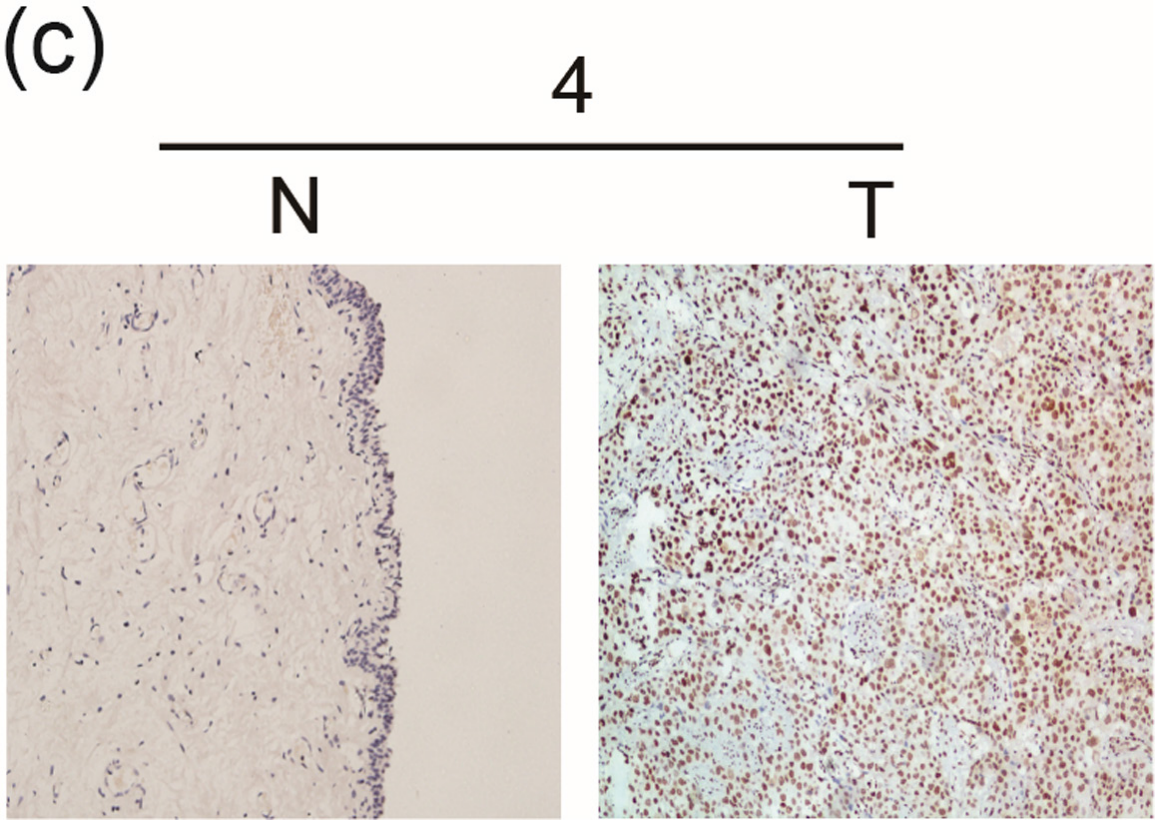
2

**Figure:**
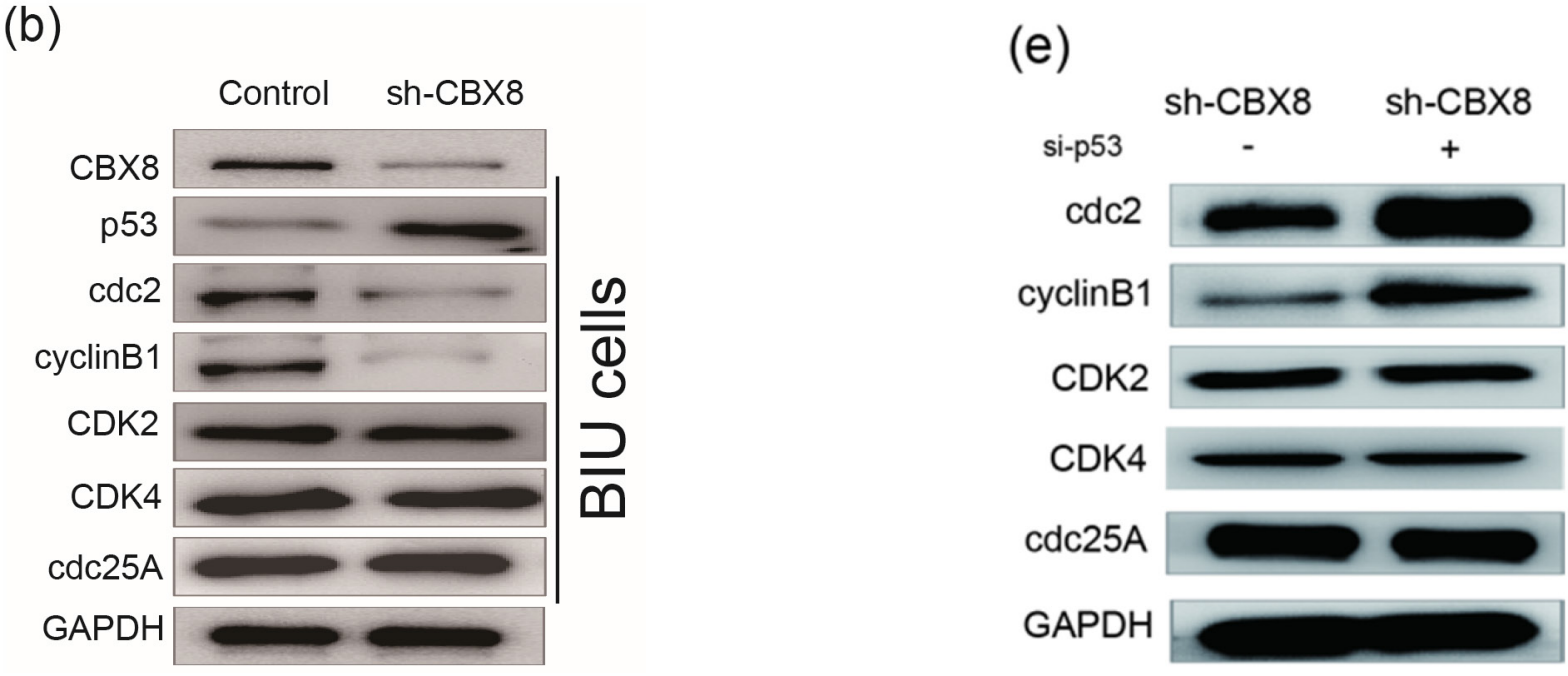
5

The authors apologize for the errors.
